# Ursodeoxycholic Acid in Patients With Treatment-Resistant Schizophrenia Suffering From Coronavirus Disease 2019: A Hypothesis Letter

**DOI:** 10.3389/fpsyt.2021.657316

**Published:** 2021-04-14

**Authors:** Mohsen Khosravi

**Affiliations:** Department of Psychiatry and Clinical Psychology, Zahedan University of Medical Sciences, Zahedan, Iran

**Keywords:** coronavirus disease 2019, patient, schizophrenia, treatment, ursodeoxycholic acid

Schizophrenia is a disabling condition with three main domains of positive, negative, and cognitive symptoms, which involves about 0.3–0.7% of the general population (1). Treatment-resistant schizophrenia (TRS) is a significant challenge in one-third of patients with schizophrenia, placing a heavy burden on families, society, and health-care professionals (2). Moreover, if these patients are infected with severe acute respiratory syndrome coronavirus 2 (SARS-CoV-2), medical treatment and psychosocial support systems will experience an increasing burden of treatment (3). Recent studies have shown that patients with schizophrenia may be vulnerable to SARS-CoV-2 infection for various reasons, including (i) poor insight and lack of decision-making capacity and effective coping skills, which lead to violation of the protective measures for inhibiting infection (e.g., physical distancing, using facial masks, and washing hands); (ii) high prevalence of comorbid metabolic disorders (more than 70%) such as hypertension, diabetes type II, coronary heart disease, and chronic pulmonary disease; (iii) high prevalence of smoking (50–90%); and (iv) homelessness or institutionalization (4). Although clozapine possesses more clinical superiorities than other antipsychotic medications in cases of TRS, its use during the viral pandemic is especially challenging due to the overlap between clozapine-induced side effects and the coronavirus disease 2019 (COVID-19) symptoms and sequelae, as well as practical difficulties in regular laboratory monitoring (5, 6). Accordingly, in the context of the COVID-19 crisis, new and effective therapeutic strategies need to be developed to treat and rescue these patients. Based on the current evidence, ursodeoxycholic acid (UDCA) seems to be an effective strategy in decreasing inflammation and avoiding cell death for patients with TRS infected with SARS-CoV-2. UDCA (C_24_H_40_O_4_) is a less toxic epimer of chenodeoxycholic acid in humans, which includes 1–5% of total bile acid components (7). Recent studies have revealed that UDCA and its main conjugate (i.e., glycoursodeoxycholic acid), as cholesterol-derived bile acids, can have neuroprotective and homeostatic properties due to their capacity to inhibit glutamate release (8, 9). Although the mechanism of the UDCA effect has not been wholly specified yet, a recent case report (10) showed that this medication could be an effective therapeutic strategy for patients with TRS. Based on the glutamate hypothesis of schizophrenia models, some of the probable therapeutic mechanisms would include (i) a decrease in the tone of glutaminergic projection neurons and, in turn, understimulation of inhibitory GABA interneurons in the ventral tegmental area, leading to the activation of mesocortical dopamine pathway, an increase of adequate dopamine release in the prefrontal cortex, and reduction of negative and cognitive symptoms of schizophrenia (11); and (ii) a decrease in firing of cortical glutaminergic projection neurons that causes an improvement in positive symptoms of schizophrenia through dopamine mesolimbic pathway hypoactivation (11, 12). Moreover, recent studies demonstrated the beneficial action of UDCA in respiratory diseases as an antioxidant, anti-inflammatory, immunomodulatory, and anti-apoptotic agents (13, 14). Some of these beneficial effects are as follows: (i) airway remodeling through the efficient modulation of Th-2-derived cytokines and inhibition of apoptosis of airway epithelial cells (15) and (ii) provoking alveolar fluid clearance in lipopolysaccharide-induced pulmonary edema via ALX/cAMP/PI3K pathway, which results in the improvement of acute respiratory distress syndrome (16). In this regard, Abdulrab et al. (17) and Subramanian et al. (18) have assumed that UDCA may have promising therapeutic effects on COVID-19-induced pneumonia and related lung edema. Bile acid derivatives have also proved to be effective in preventing virus entry by reducing the adhesion of SARS-CoV-2 spike's protein receptor binding domain to its angiotensin-converting enzyme 2 consensus *in vitro* (19). In addition, Yadav et al. (20) suggested that chenodeoxycholate and ursodeoxycholate can be potential candidates to hinder the survival of SARS-CoV-2 via disrupting the structure of envelope protein of SARS-CoV-2 and facilitating the entry of solvents/polar inhibitors into the viral cell. Interestingly, Rigamonti et al. (21) recently found quite low rates of symptomatic SARS-CoV-2 infection in patients with biliary cholangitis treated with UDCA. In total, these findings may highlight the emergence of novel therapeutic strategies against COVID-19 among patients with TRS. However, further studies are needed, particularly regarding efficacy and tolerability of UDCA in this group of patients.

## Author Contributions

MKH conceptualized the opinion and wrote the manuscript. The author also approved the final version of the submitted manuscript.

## Conflict of Interest

The author declares that the research was conducted in the absence of any commercial or financial relationships that could be construed as a potential conflict of interest.
